# Geographical peer effect in serial mergers and acquisitions: The influence of social learning and director network

**DOI:** 10.1371/journal.pone.0294950

**Published:** 2023-12-21

**Authors:** Xiaoxu Zhang, Yu Song, Qiaoran Liao

**Affiliations:** School of Business Administration, University of Science and Technology Liaoning, Anshan, China; East China Normal University, CHINA

## Abstract

Based on the serial mergers and acquisitions(M&A) data of Chinese A-share listed companies from 2010 to 2019, this paper discusses whether there are geographical peer effects in serial M&A, and tests its mechanism and influence path. The empirical study shows that geographical peer decision-making has geographical peer effects in the decision-making of serial M&A, and the average number of serial M&A of geographical peers has a significant positive impact on the serial M&A decision-making of focal firms. Social learning and director networks are essential to produce geographical peer effects in serial M&A. The external learning mechanism shows that the geographical peer effect of serial M&A decision-making is partly caused by backward firms’ social learning to the leading peer firms’ decision-making, but vice versa. The director network relationship of focal firms can positively moderate the relationship between the geographical peer firms’ average serial M&A decision-making and the focal firm’s serial M&A decision-making. This paper reveals the mechanism of geographical peer effect in serial M&A decision-making and provides a new perspective to understand the motivation of serial M&A decision-making. These empirical findings not only provide important empirical evidence on serial M&A from developing countries such as China, but also provide a valuable reference for decision-makers and researchers of serial M&A in the future.

## Introduction

M&A is an essential means to optimize the allocation of corporate resources and stimulate the vitality of firms [1–3], and can help firm improve their debt structure [4, 5], optimize their organizational structure [4, 6], promote their technological innovation [7–9], and improve their performance [6, 10, 11], which are strategically crucial for their high-quality development. According to the number of mergers and acquisitions by firms over a period, M&A can be classified into single M&A and serial M&A. However, most existing literature ignores the difference between single and serial M&A, regards M&A transactions as independent events [12], and pays little attention to serial M&A. The study of serial M&A is first proposed by Schipper and Thompson(1983) [13] and expressed as “acquisition programs”. It is emphasized that a single M&A is a part of “acquisition programs” carried out by a firm over a period rather than an isolated or unrelated M&A event. Fuller, Netter and Stegemoller (2002) [14] have found that the returns of the first M&A were significant, then the returns of M&A decreased gradually, and even after the fifth M&A, scholars began to pay more and more attention to the problem of serial M&A. However, the existing research on serial M&A is still limited. The concrete manifestation is that we do not pay enough attention to the informational characteristics of each M&A transaction in serial M&A. In particular, there is little literature to explore the influencing factors and mechanism of serial M&A decision-making based on the perspective of peer firms’ decision-making. Based on the internal perspective of the firm, the existing research has explained the motivation of the serial M&A from the perspective of technology acquisition [15], synergy effect [16] and merger experience [17–20]; Based on the perspective of corporate managers, existing studies have investigated the motivations of the serial M&A from the perspective of managerial compensation [21], principal-agent conflict [22–24], self-interest motivation [22, 25] and overconfidence [26–28]. Based on the perspective of the external environment, existing research has explained the motivations of serial M&A from the perspective of industrial structure adjustment [29], economic policy uncertainty [30] and opportunity set attraction [31]. The above studies mainly focus on the study of serial M&A based on the firm’s characteristics, ignoring that serial M&A may also be affected by the serial M&A of peer firms [32].

In recent years, the peer effect in corporate decision-making has begun to attract the attention of scholars. Unlike the traditional theories that only focus on the individual characteristics of firms, peer effect theory holds that the influence relationship between peer firms can effectively explain the preferences of decision-makers. The study has found that the choice of capital structure [33], social responsibility participation [34], green innovation [35], compliance with extortive requests [36], corporate environmental information disclosure [37], management earnings forecasts [38] and M&A target selection [39] are all affected by peer effect. “Peer Effect” refers to the phenomenon that individual behavioral decision-making is influenced by the decision-making of related peers. As one of the focuses that scholars pay close attention to, the concept of peer effect does not have a uniform definition standard. As long as there is a specific interactive potential influence between equal individuals in social relations, this effect can be called the peer effect. When examining that a firm is affected by other firms in the reference group (geography or industry), the firm is called the focal firm, and the other firms are called (geographical industrial) peer firms.

Previous studies on the peer effects of corporate decision-making have focused on the decision-making imitation among peer firms in the industry. This undoubtedly provides an opportunity for this study. First, in addition to firms in the same industry, firms in the same geography are often selected as target firms to imitate, thus forming the geographical peer effect. Geographical conditions such as the level of economic development, financial development and the degree of market-oriented competition may cause firms to imitate local leading firms or related firms to make decisions. Significantly when the above conditions change, the future uncertainty firms face increases, so it is more necessary to find a learning or imitation object that can be referred to. When the distance between peer firms is significant, decision-making information needs to go through more paths to reach the focal firm, which increases the cost of capturing and searching for information. The proximity of the distance between peer firms in the same geography is conducive to the flow of decision-making information between firms, and the proximity of distance is more helpful for the focal firms to obtain privileged information [40, 41]. Firms in the same geography have the same culture, customs, values and codes of conduct, and these unique attributes can protect the proprietary information from being exchanged only among peer firms [42]. Therefore, the same geographical peer firms can avoid the impact of M&A rumors on decision-making [43], make firms share decision-making information with closely related geographical peer firms, and make it difficult for firms outside the province to access and obtain relevant information [40].

Second, most of the existing studies on the driving factors of corporate serial M&A only focus on the internal and managerial factors, among which the internal factors include efficiency drivers, resource dependence drivers, market mispricing drivers, etc., and managerial factors Including managers’ opportunistic motivation, managers’ overconfidence motivation, etc., ignoring the external influence of corporate peers’ serial M&A decision-making.

Third, in practice, the decision-making of serial M&A has great uncertainty, and the research of this paper has important guiding significance for understanding the serial M&A decision-making relationship among geographical peer firms in China. Under the uncertain economic policy environment and incomplete and asymmetric investment information, the geographical peer effect is common [44]. In the Chinese capital market, many examples of social interaction among geographical peer firms, groups, and individuals observe, imitate, and learn from each other, resulting in consistent behavioral decisions among peers. Taking China’s express delivery industry as an example, YTO, STO, ZTO, and Rhyme, which account for half of China’s private express delivery industry, all come from Tonglu County, Zhejiang Province, and Tonglu County is therefore known as the “hometown of express delivery”. Taking Chinese sports shoe brands as an example, Fujian, as the “shoe capital of Jinjiang”, has produced original brands such as Hongxing Erke, Xtep, Anta, 361, Peak and Fuguiniao. Take Yanling lighting engineers, who have influenced the film and television industry, as an example. 80% of the Chinese film and television industry’s lighting practitioners and equipment rental companies are from Yanling County, Henan Province. It can be seen that the geographical peer effect of firm, group and individual decision-making has become the norm, so the existence of geographical peer effect will challenge the independence of corporate decision-making. Will the serial M&A decision-making of geographical peer firms influence each other? In the case of differences in the status advantages of firms in the same region, whether the “backward” focal firms or “leading” focal firms are more willing to imitate the serial M&A decision-making of peer firms? In the case of rich social network relationships among geographical peer firms, should focal firms choose to imitate peer firms or maintain decision-making independence? In this context, it is particularly important to study the geographical peer effect of serial M&A decision-making.

This paper intends to use the data of Chinse-listed companies from 2010 to 2019 to empirically analyze the mechanism and path of geographical peer effects affecting corporate serial M&A decisions. From the perspectives of “social learning” and “director network”, this paper attempts to reveal the complexity of geographical peer effect in the process of serial M&A decision-making, and to explain the difference between learning from leading peers and learning from backward peers, and how the director network relationship enhances the impact of geographical peer effect on serial M&A decision-making. In addition, this study also examines the heterogeneous effects of focal firm’s serial M&A, peers’ average serial M&A and internal learning on geographical peer effect and serial M&A decision-making relationship. The research conclusions have important theoretical and practical implications for understanding how the geographical peer effect affects the decision-making of serial M&A and the scientific choice of the decision-making of serial M&A.

The possible contributions of this article are as follows: Firstly, it enriches and expands the analytical perspective of serial M&A decision-making. The traditional research explains corporate serial M&A decisions from theoretical perspectives such as synergistic effect theory, market power theory, and principal-agent theory. The geographical peer effect that this study focuses on expands the understanding and understanding of serial M&A, breaks through the bottleneck of the traditional theory that pays attention to serial M&A only from the corporate internal characteristics and the characteristics of managers, and is helpful to understand the corporate serial M&A decisions close to the situation in China; Secondly, it supplements the identification method of geographical peer effect of serial M&A. The geographical peer effect of serial M&A is identified by using the average number of serial M&A of other peer firms in the same region, and the multiple robustness test is carried out, which is helpful to improve the validity of measuring the variables of geographical peer effect of serial M&A. This paper provides a reference for comprehensively identifying the geographical peer effect of serial M&A; Thirdly, it makes a valuable exploration of the mechanism and path of the geographical peer effect. Different from the blind imitation of the “herding effect”, this paper clarifies that the geographical peer effect of serial M&A is the rational learning of peer leaders. This paper also explains the formation mechanism of geographical peer effects from the perspective of the director network.

## Theory and hypothesis

### The geographical peer effect in serial M&A

Traditional financial theories usually assume the independence of corporate decision-making but pay little attention to the interactive impact of peer firm decision-making. As the peer effect has been widely studied in the fields of pedagogy [45–47], social psychology [48] and social economics [49, 50], the impact of peer effect in corporate decision-making has gradually attracted the attention of financial scholars. Due to the uncertainty of the external environment and the asymmetry of information between firms, the decision-making of serial M&A is faced with more significant uncertainty. Firms in the same province or region face similar economic environments, legal systems, market competition pressure, cultural customs, and language. Focal firms can imitate the serial M&A decision-making of their peer firms through face-to-face communication to reduce the uncertainty of M&A decision-making [51]. In different provinces or regions, due to the differences in the level of economic development, financial development, market-oriented competition, social culture and so on, the serial M&A activities of firms are also different, which makes the serial M&A decision-making between firms different among regions. Almazan, Motta and Titman et al. (2010) have studied the relationship between the firm’s geographical location and M&A opportunities, and found that firms located in industrial clusters face more M&A opportunities [52]. Gao, Ng and Wang (2011) have studied the influence of geographic location on the decision-making of corporate capital structure, and found that firms in the same region showed similar financial decisions, and believed that the geographical location of firms could explain the differences in capital structure of cross-regional firms [53]. Dougal, Parsons and Titman (2015) have analyzed the peer effect on investment among firms in the same region from the perspectives of knowledge and technology spillover effects, consumption externalities, infrastructure construction, and mortgage value [54]. Therefore, based on the existing research, this paper uses information-based theory, rivalry-based theory, culture and language to establish the research hypothesis that the serial M&A decision-making of Chinese listed companies has geographical peer effects.

First, information-based theory. Although referring to the M&A experience of focal firms may be the priority for serial M&A decision-making, existing research has shown that due to the limitations of M&A time interval and information matching, the firm’s own M&A experience cannot guarantee the effectiveness of serial M&A decisions [12, 55, 56]. In this case, firms will look for other sources of empirical information. Among them, imitation is a low-cost strategy to alleviate information asymmetry and reduce the risk of competition. When the uncertainty is high, the focal firms tend to obtain strategies to deal with the uncertainty from peer firms. According to Lieberman and Asaba (2006) [57], corporate imitation is classified into information-based and rivalry-based theories. The information-based theory means that the focal firm follows the decision-making of the other peer firms, which are considered to have better information. According to the information-based theory, decision-makers with limited information can accumulate experience and obtain information by learning and imitating the serial M&A decision-making of geographical peer firms, to reduce the uncertainty of decision-making results. Kaustia and Rantala (2015) [58] pointed out that imitating the decision-making of peer firms will not bring direct benefits, but failure to do so will lead to a series of problems. For example, when other peer firms in the same region implement serial M&A decision-making, but the focal firm does not choose to implement M&A, it may lose part of its resource advantages in the long run. Therefore, the focal firm obtains empirical information by imitating the serial M&A decision-making of geographical peer firms, which will help to reduce the time, cost and risk spent on serial M&A decisions.

Second, rivalry-based theory. There are potential competitive pressures among firms with similar resource endowments. Although differentiation strategies can be adopted to resolve competitive pressures, differentiation strategies are often complicated and risky. Therefore, to maintain a competitive position or alleviate competitive pressure, the focal firm usually imitates the decision-making of peer competitors. The rivalry-based theory implies that the focal firm imitates the decision-making of the other peer firms in order to maintain equal competition or restrict competition. According to the rivalry-based theory, the more similar the resource endowment among firms, the more tremendous competition pressure on each other in the market. Imitation shows the attitude of firms to defend the status quo, neither giving up their current market position nor falling into a disgusting competition that destroys each other [32]. The research of Adhikari and Agrawal (2018) [59] has shown that in order to alleviate the competitive pressure in the fierce market environment, focal firms have a solid motivation to learn and imitate the decision-making of peer firms to avoid falling behind. Effective serial M&A decision-making can enhance the market value of firms, and relate to the future development direction of firms. However, when the decision-making of serial M&A fails, the firm may suffer property losses and risk bankruptcy. Therefore, the focal firm has a solid incentive to adjust its decision-making according to the serial M&A decision-making of geographical peer firms, to minimize the competitive pressure and the risk of decision-making failure.

Third, culture and language. Social identity theory holds that cultural similarity can establish and maintain social identity among organizations. Culture potentially impacts individual habits, preferences, and risk attitudes [60–62], affecting firms’ cognition, interaction, and decision-making choices [63, 64]. DiMaggio (1997) has pointed out that cultural similarity affects communication and cooperation between individuals. Individuals prefer “similar attraction” in the communication process, and learn from objects with similar social status because similar objects come with similar resources, opportunities and obligations. Culture leads to differences in behavioral decision-making among individuals by influencing individuals’ perceptions of basic facts [65] and attitudes towards risks [66, 67]. It can be seen that the more similar the behavior, culture, etc., among firms, the easier it is to have a strong sense of identity with specific strategies [68]. Language is the first crucial cultural representation in interpersonal communication. The same dialect will form a network connection between individuals, which can improve the intimacy and social identity between members [69]. In addition, language can be regarded as the accumulation of knowledge capital [70], has a capital attribute, and may increase or decrease transaction costs between firms [71, 72]. Hong and Stein (2007) [73] have found that the same information may be interpreted differently in different languages, which increases the cost of information exchange to a certain extent. Sharing language, contextual interpretation and cultural customs can reduce the differences caused by implicit decision-making information transmission and the communication cost caused by cultural differences [74]. The proximity of culture, language and geographical location can promote the homogenization of cognition among firms. In particular, China is a typical “nepotist society”, and peer firms with close culture, language and geographical location are more likely to form cognitive homogenization. Individuals with the same culture will form an “intergroup”, while others with different cultures will be classified as “outgroup”. Individuals tend to allocate favorable resources to intergroup members and give them more positive evaluations while assigning fewer resources and negative evaluations to outgroup members [75]. Research by Dougal Parsons and Titman (2015) [54] has shown that the efficiency of information transmission and sharing among peer firms in the same region is higher than other firms in the same region. It is more helpful for firms to obtain private information and tacit knowledge, thereby forming the similarity and convergence of peer firms’ decision-making. Due to different geographical environments, historical inheritance and economic development, different regions of China have formed massive differences in cultural and linguistic characteristics. This multi-level cultural and dialect difference will inevitably affect the serial M&A decision-making activities of firms. Therefore, this paper holds that under the influence of the same culture and language, the lower the communication cost among peer firms in the same region and the stronger the sense of social identity, the easier it is to form the same serial M&A decision-making, thus producing the geographical peer effect in serial M&A decision-making.

To sum up, there are apparent similarities and convergences in the decision-making of serial M&A among firms in the same region, showing the geographical peer effect of serial M&A. Based on the above analysis, the research hypothesis is put forward as follows:

H1: There is a geographical peer effect in corporate serial M&A decision-making.

### The impact of social learning

Imitation learning is an essential way of behavioral learning. Focal firms observe the behavior of other firms and implement similar behaviors [76–78]. According to social learning theory, focal firms learn from the experiences of other firms through imitation, and bring external resources, information and knowledge into the firm through imitation, and bring external resources, information and knowledge into the firm through imitation and apply them. The starting point of social learning is to identify imitation objects of external heterogeneous information. Considering the uncertainty of decision-making and the cost of obtaining relevant information, managers can learn from other peer firms in the same province or region. So, which peer firms are the imitation learning objects of the focal firms? Large-scale firms usually have a more mature organizational structure, more skilled managers, rich experience in mergers and acquisitions, more valuable expertise and contacts, and occupy a certain leading position in the market. Firm size is a firm fundamental variable, and even slight differences may have a key impact on dependent variables and other independent variables in empirical research. Within the geo-reference group, the generation of geographical peer effect in serial M&A decision-making may vary with the firm size.

According to the law of logical imitation proposed by Trade (1903) [79], the selection of imitation objects and the degree of imitation conform to the inherent logic law; that is, those leading firms with large scale and high status are more likely to become imitation objects and to be imitated. Foucault and Fresard (2014) [80] have constructed a theoretical model of learning on the peer effect of investment decision-making, and the results showed that learning behavior is one of the reasons for the peer effects of a stock split. Kaustia and Knüpfer (2012) [81] have shown that the reason why new investors are willing to enter the stock market is mainly due to the observation that their peers enjoy higher-than-average portfolio returns. Kaustia and Rantala (2015) [58] have found that focal firms are more likely to split shares when peer firms conduct stock splits, which is consistent with social learning of peer firm behavior and outcomes. Banerjee (1992) [82], Bikhchandani, Hirshleifer and Welch(1998) [83] have believed that managers will rely more on the information of other firms to make decisions, especially those of leading firms when the cost of obtaining information is high, or the noise of information they have is high, large-scale, more successful or more prestigious firms are more likely to be imitated [57, 83, 84]. Bursztyn, Eder and Ferman et al. (2014) [85] have tested the effect of social learning on the peer effect mechanism through two sets of data of “sophisticated” market investors and “young” market investors, and found that the former has a weak peer effect. The latter has a more substantial investment peer effect. According to the above research, due to the differences in scale, reputation and maturity, the abilities of all kinds of firms to obtain useful information are different. “backward” focal firms have a strong motivation to learn decision-making from their peers because of their low information quality. However, “leading” focal firms have relatively weak motivation to learn from their peers because of their high information quality.

It can be seen that leading firms with information advantages tend to make independent decisions, while backward firms without information advantages are more inclined to imitate and follow. According to the information-based theory, large-scale firms have richer M&A information sources, knowledge accumulation, capital and other resources, and are more autonomous in making serial M&A decision-making. However, the resource acquisition channels of “backward” firms which with small-scale are relatively simple, and the risks and costs of hasty decision-making are relatively high. Therefore, they are more inclined to imitate the serial M&A decision-making of “leading” firms. At the same time, according to the theory of competitive imitation, “leading” firms occupy a dominant position in the market, and their M&A behavior will further enhance their market power, so other peer firms are more likely to imitate their serial M&A decision-making to narrow the market power gap. Therefore, to strengthen the legitimacy of firm imitation learning within the same region, “leading” firms are more likely to be imitated, and “backward” firms are more likely to be followers. Based on the above analysis, this paper puts forward the following assumptions:

H2a: The “backward” focal firms have more significant geographical peer effect in serial M&A.
H2b: The “leading” focal firms have a weak geographical peer effect in serial M&A.

### The impact of director network

The social network is trust, structure, and cognition established by social solidarity, and can bring various resources and benefits to individuals in the network relationship [86]. In the peer effect of individual decision-making, the social network plays an important role; that is, by extending the information exchange channels among peers, the social network breaks the independence of individual decision-making, and then affects the decision-making preference of individuals. For example, individual decisions are often influenced by neighbors, classmates, colleagues, etc.[87]. The “Transfer to Opportunity” social experiment has investigated the neighborhood social network and has found that neighbors influence individual behavior outcomes, and neighbors with higher socio-economic status will have positive externalities on the individual economy, health, and safety [88]. Sacerdote (2011) [89] tested the peer effect on students and found that the existence of unique social network relationships such as race, political attitude, drinking, and criminal behavior has a more significant impact on students than on their grades.

The director network is an essential form of corporate social network relationship, which is established, connected, and formed through the concurrent appointment of directors among firms. The widespread existence of the director network promotes the close integration of the firms and the capital market. Granovetter (1973) [90] has pointed out in “The Strength of Weak Ties” that social network is a bridge connecting macro behavior and micro behavior. From the macro perspective, the director network can be regarded as a collection of network groups with specific characteristics that can be distinguished by organizational culture, behavioral norms, reliable environment, etc. Different unique resources make members from different director networks express different characteristics, which are also reflected in the decision-making of members. The privacy, importance and reliability of the information obtained by the director network is an important information channel between firms, and essential information about corporate financial decision-making can be disseminated between the networks [91]. The more relationships the director network can connect, the more frequent social interactions between geographical peer firms will be. The more information access channels the focal firms have and the lower the cost of information acquisition, which makes the stronger the willingness of the focal firm to learn, imitate or follow the geographical peer firms to make decisions during serial M&A. From the micro perspective, the member behavior of the director network is affected by the network group structure. Its decision-making is influenced by the influence of other members in the network and the nepotism generated by the network connection, which deviates from the subjective initiative. For those firms with rich network relationships of directors, the experience accumulated by the directors enables the focal firms to make M&A decisions by learning and imitating other network member firms [92]. The closer the distance between network members, the more it helps members to obtain the privilege, novelty and diversity of information [40]. Studies have shown that network connections have changed the interaction criterion among the network members from being based on fair trade to being based on nepotism [93]. This nepotism can be used as an information protection mechanism so that information is only shared among closely related local firms. At the same time, it is difficult for non-local firms to access, avoiding disclosing effective information and disseminating invalid information [40, 43, 94]. In Chinese nepotist society, peer firms embedded in the director network identify. It enhances the efficiency of information exchange among firms, optimizes the judgment on the risks and opportunities of serial M&A, and thus strengthens the geographical peer effect of serial M&A decision-making. Based on the above analysis, the hypothesis is put forward as follows:

H3: The richer the director network relationship is, the stronger the geographical peer effect in serial M&A.

**Fig 1:**
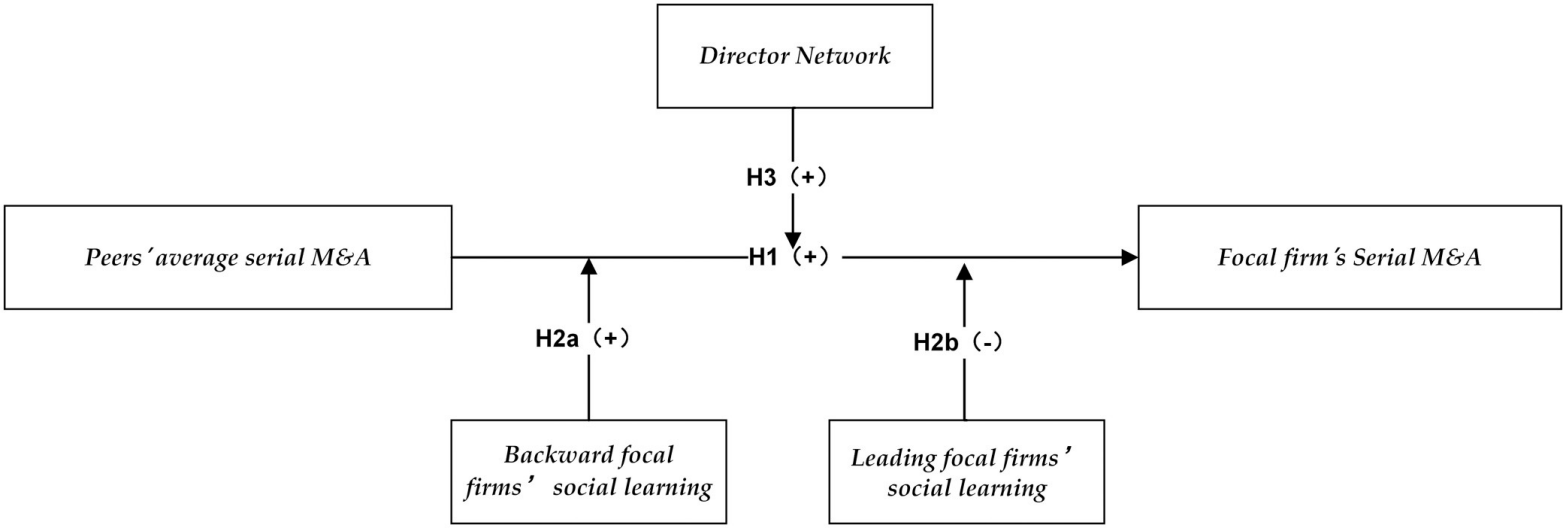
The research framework of peer effect in serial M&A.

In summary, the research framework of this paper is shown as Fig 1.

## Materials and methods

### Sample selection

According to the data availability of serial M&A, the M&A samples selected in this paper are M&A transactions with the first announcement date of the M&A companies in 2010–2019. The screening criteria for M&A events are as follows: (1) Select the sample of Chinese listed companies with successful M&A transactions; (2) exclude samples of related M&A transactions because related M&A may have a strong motivation for earnings management; (3) exclude samples of the financial industry and ST companies; (4) exclude the sample of companies with a transaction value of fewer than 1 million yuan, because the M&A with a small transaction amount does not have a significant impact on the listed company; (5) In order to keep the sample of M&A events clean, only the sample with the largest transaction amount is retained for multiple M&A in the same company with the same date of the first M&A announcement; (6) exclude the samples of single M&A; (7) exclude samples with asset-liability ratios greater than 1 or less than 0, and cash holdings greater than 1, because the operating conditions of companies with such extreme values fluctuate considerably. Finally, 14 519 sample data are obtained. The definition of geographical peer firms in this paper is based on the location of firm registration, the 31 provinces (autonomous regions, municipalities directly under the Central Government) in mainland China. The data in this paper are selected from the CSMAR and WIND database, and STATA14.0 is mainly used to complete the statistical analysis of the data.

### Empirical model

In order to test H1, regarding the study of Manski(1993) [95], the model for testing the geographical peer effects in serial M&A is as follows:

$$\begin{array}{c} {SMA}_{i,t}=\alpha_{0}+\alpha_{1}\bar{{SMA}_{-i,t}^{\alpha }}+\alpha_{i}\sum Control_{i,t}+\epsilon \end{array}$$ (1)

Where the subscript *i* represents the focal firm, the subscript *t* represents the year, and the subscript *α* represents the region. *SMA*_*i*,*t*_ is the explained variable, which represents the number of corporate serial M&A in the year; $\bar{{SMA}_{-i,t}^{\alpha }}$ is the explanatory variable, which represents the average number of serial M&A of the other peer firms (except the focal firm) in the region *α* in the year *t*. ∑*Control*_*i*,*t*_ is the control variable group, consisting of 7 variables. *α*_0_, *α*_1_ and *α*_*i*_ are the parameters to be estimated. Model (1) mainly focuses on the estimation results of *α*_*i*_, if it is significant and greater than 0, it indicates that there is geographical peer effect in corporate serial M&A. *ϵ* represents the residual.

In order to test H2a and H2b, according to the ranking of firms in the same region according to their asset size, this paper divides the samples into “leading” firms and “backward” firms. It sets the model for testing the impact of social learning on the geographical peer effect of corporate serial M&A as follows:

$$\begin{array}{c} SMAbot_{i,t}^{\alpha }=\beta_{0}+\beta_{1}\bar{SMAtop_{-i,t}^{\alpha }}+\beta_{i}\sum Control_{i,t}+\epsilon \end{array}$$ (2)

$$\begin{array}{c} SMAtop_{i,t}^{\alpha }=\chi_{0}+\chi_{1}\bar{SMAbot_{-i,t}^{\alpha }}+\chi_{i}\sum Control_{i,t}+\epsilon \end{array}$$ (3)

Models (2) and (3) are based on model (1) and grouped according to the corporate asset size. $SMAbot_{i,t}^{\alpha }$ and $SMAtop_{i,t}^{\alpha }$ are explained variables representing the backward focal firm’s serial M&A and leading focal firm’s serial M&A, respectively. $\bar{SMAtop_{-i,t}^{\alpha }}$ and $\bar{SMAbot_{-i,t}^{\alpha }}$ are explanatory variables, respectively represent the leading peers’ average serial M&A and backward peers’ average serial M&A. *β*_0_, *β*_1_, *β*_*i*_, *χ*_0_, *χ*_1_ and *χ*_*i*_ are parameters to be estimated. Model (2) mainly focuses on the estimation results of *β*_1_, if *β*_1_ is significant and greater than zero, indicating that the peer effect is partly derived from the rational learning of the “backward” focal firms from the geographical “leading” peer firms’ serial M&A decision-making. Model (3) mainly focuses on the estimation results of *χ*_1_, if *χ*_1_ is significant and greater than zero, indicating that the peer effect is partly due to the irrational learning of serial M&A decision-making by “leading” focal firms to geographical “backward” peers companies.

In order to test H3, this paper sets the model for testing the influence of director network relations on the geographical peer effect of corporate serial M&A as:

$$\begin{array}{c} \begin{array}{cc} {SMA}_{i,t}^{\alpha } & =\delta_{0}+\delta_{1}\bar{{SMA}_{-i,t}^{\alpha }}+\delta_{2}Network_{i,t}+\delta_{3}\bar{{SMA}_{-i,t}^{\alpha }}\times Network_{i,t}\\ & +\delta_{i}\sum Control_{i,t}+\epsilon \end{array} \end{array}$$ (4)

Model (4) is based on Model (1) by adding moderator variables director network relationship *Network*_*i*,*t*_ and the multiplication term $\bar{{SMA}_{-i,t}^{\alpha }}\times Network_{i,t}$ of geographical peer effect and director network relationship. *δ*_0_, *δ*_1_, *δ*_2_, *δ*_3_ and *δ*_*i*_ are parameters to be estimated. Model (4) mainly focus on the estimated coefficient *δ*_3_. If *δ*_3_ is significant and greater than zero, indicating that *Network*_*i*,*t*_ is an effective moderator variable, that is, the network relationship of directors strengthens the influence of the geographical peer effect in the corporate serial M&A.

### Variable

Explained variables: *SMA*_*i*,*t*_ is the total number of serial M&A of the focal firm *i* in the year *t*; $SMAbot_{i,t}^{\alpha }$ is the total number of serial M&A of the focal firm *i* which is in the bottom 90% of the asset rankings in the province in the year $t.SMAtop_{i,t}^{\alpha }$ is the total number of serial M&A of the focal firm *i* which is in the top 90% of the asset rankings in the province in the year *t*.

Explanatory variable: $\bar{{SMA}_{-i,t}^{\alpha }}$ is the average value of serial M&A of geographical peer firms, expressed as the average value of the total number of serial M&A of all firms in the province where the focal firm *i* is registered in the year *t* minus the number of serial M&A of the focal firm. $SMAtop_{i,t}^{\alpha }$ is the average value of serial M&A of the geographical leading peers, which is the average number of serial M&A of the top 10% of the assets of serial M&A in the same province *α* in the same year *t*, except for the focal firm *i*. $\bar{SMAtbot_{-i,t}^{\alpha }}$ is the average value of serial M&A of the backward peers, which is the average number of serial M&A of the bottom 10% of the assets of serial M&A in the same province *α* in the same year *t*, except for the focal firm *i*.

Moderator variable: *Network*_*i*,*t*_ is the director network relationship of the focal firm *i* in year *t*, which is measured according to the richness of the structural holes formed by director connections in firm *i*. According to the appointment information of directors, the PAJECK analysis software is used to convert the 2-mode matrix established between directors and firms into a 1-mode matrix between firms to reveal the director network relationship of the focal firms. Referring to the practice of Zaheer and Bell (2005) [96], the calculation method of is shown in model (5).

$$\begin{array}{c} Network_{i,t}=1-\sum_{i\neq c}{\left[D_{ic}+\sum_{b\neq i,c}\left(D_{ib}D_{bc}\right)\right]}^{2} \end{array}$$ (5)

Where *D*_*ic*_ represents the strength of the direct relationship between the focal firm *i* and firm *c*; *D*_*ib*_*D*_*bc*_ represents the strength of the indirect relationship between the focal firm *i* and firm *c*, and this indirect relationship is connected by the bridge enterprise *b*. The sum of direct and indirect relationship strengths of the focal firm *i* represents the closure of network relations and the lack of structural holes in the firm. The restrictive index difference between 1 and the structural hole of the firm represents the richness of the director network structural hole.

Control variables: The design of control variables selects the firm size(*Size*_*i*,*t*_), financial leverage(*Lev*_*i*,*t*_), cash(*Cash*_*i*,*t*_), shareholding(*Hold*_*i*,*t*_), and boards(*Board*_*i*,*t*_). Table 1 presents the definitions of the main variables.

**Table 1:** Definitions of the main variables.

Variable	Definitions		
Explained	focal firm’s serial M&A	*SMA*_*i*,*t*_	sum of M&A that occurred in focal firm *i* in year *t* following Zhang,Yao and Du(2021) [32]
	backward firm’s serial M&A	$SMAbot_{i,t}^{\alpha }$	sum of M&A of the focal firm in the bottom 90% of the asset ranking in province *α* in year *t* following Fu,Yang and Fu(2015) [97]
	leading firm’s serial M&A	$SMAtop_{i,t}^{\alpha }$	sum of M&A of the focal firm in the top 90% of the asset ranking in province *α* in year *t* following Fu,Yang and Fu(2015) [97]
Explanatory	peer’s average serial M&A	$\bar{{SMA}_{-i,t}^{\alpha }}$	average of M&A of peer firms in the same province *α* in year *t* following Zhang,Yao and Du(2021) [32]
	leading peer’s average serial M&A	$\bar{SMAtop_{i,t}^{\alpha }}$	average of M&A of peer firms in the top 10% in province *α* in year *t* following Fu,Yang and Fu(2015) [97]
	backward peer’s average serial M&A	$\bar{SMAbot_{i,t}^{\alpha }}$	average of M&A of peer firms in the bot 10% in province *α* in year *t* following Fu,Yang and Fu(2015) [97]
Moderate	director network	*Network*_*i*,*t*_	refer to Model (5) following Zaheer and Bell (2005) [96]
Control	firm size	*Size*_*i*,*t*_	natural logarithm of total assets of focal firm *i* in year *t* following Moeller, Schlingemann and Stulz(2004) [22]
	financial leverage	*Lev*_*i*,*t*_	Ratio of total liabilities to total assets following Maloney, McCormick and Mitchell(1993) [98]
	cash	*Cash*_*i*,*t*_	cash flow of operating activities divided by average total assets following Ismail(2005) [99]
	shareholding	*Hold*_*i*,*t*_	shareholding ratio of senior executives in focal firm *i* following Hayward and Hambrick(1997) [100]
	board	*Board*_*i*,*t*_	sum of board members following Capron and Shen(2007) [101]

## Results analysis

### Descriptive statistics and correlation analysis

Table 2 presents the descriptive statistics of main variables. From the mean, minimum, 25%quantile, and 75%quentile, we can see a corresponding relationship between *SMA*_*i*,*t*_ and $\bar{{SMA}_{-i,t}^{\alpha }}$. In contrast, the standard deviation of *SMA*_*i*,*t*_ is greater than that of $\bar{{SMA}_{-i,t}^{\alpha }}$,indicating that the gap in the number of serial M&A between the focal firm is greater than the average number of peer firms’ M&A. Detailed definitions of main variables are reported in Table 1.

**Table 2:** Descriptive statistics of the main variables.

*Variable*	N	Mean	Std.Dev.	Min	P25	Median	P75
*SMA*_*i*,*t*_	14519	1.136	1.928	1	0	0	2
$SMAbot_{i,t}^{\alpha }$	13118	1.094	1.676	0	0	1	2
$SMAtop_{i,t}^{\alpha }$	13099	1.156	1.968	0	0	1	2
$\bar{{SMA}_{-i,t}^{\alpha }}$	14519	1.136	0.524	0.303	0.695	1.107	1.517
$\bar{SMAtop_{-i,t}^{\alpha }}$	13118	1.507	0.942	0.056	0.667	1.444	2.149
$\bar{SMAbot_{-i,t}^{\alpha }}$	13099	0.949	0.471	0.231	0.630	0.800	1.216
*Network*_*i*,*t*_	14519	0.743	0.221	0	0.644	0.824	0.898
*Size*_*i*,*t*_	14519	22.479	1.636	18.933	21.468	22.322	23.357
*Lev*_*i*,*t*_	14519	0.505	0.225	0.069	0.337	0.504	0.664
*Cash*_*i*,*t*_	14519	0.036	0.170	0	0.001	0.042	0.085
*Hold*_*i*,*t*_	14519	0.046	0.132	0	0	0.0003	0.002
*Board*_*i*,*t*_	14519	8.946	1.987	5	8	9	9

The correlation analysis results in Table 3 show that *SMA*_*i*,*t*_ and $\bar{{SMA}_{-i,t}^{\alpha }}$ are significantly positively correlated, providing preliminary evidence for H1 of this study.

**Table 3:** The correlation analysis of main variables (N = 14519).

*Variable*	*SMA*_*i*,*t*_	$\bar{{SMA}_{-i,t}^{\alpha }}$	*Network*_*i*,*t*_	*Size*_*i*,*t*_	*Lev*_*i*,*t*_	*Cash*_*i*,*t*_	*Hold*_*i*,*t*_	*Board*_*i*,*t*_
*SMA*_*i*,*t*_	1	0.225*** *p*=0.0000	0.034*** *p*=0.0000	0.618*** *p*=0.0000	0.045*** *p*=0.0000	−0.072*** *p*=0.0000	0.194*** *p*=0.0000	−0.071*** *p*=0.0000
$\bar{{SMA}_{-i,t}^{\alpha }}$	0.209*** *p*=0.0000	1	0.001 *p*=0.9187	0.084*** *p*=0.0000	−0.108*** *p*=0.0000	0.058*** *p*=0.0000	0.217*** *p*=0.0000	−0.099*** *p*=0.0000
*Network*_*i*,*t*_	0.049*** *p*=0.0000	−0.045*** *p*=0.0000	1	0.189*** *p*=0.0000	0.106*** *p*=0.0000	-0.003 *p*=0.7395	−0.048*** *p*=0.0000	0.175*** *p*=0.0000
*Size*_*i*,*t*_	0.074*** *p*=0.0000	0.070*** *p*=0.0000	0.182*** *p*=0.0000	1	0.396*** *p*=0.0000	0.079*** *p*=0.0000	−0.021** *p*=0.0133	0.262*** *p*=0.0000
*Lev*_*i*,*t*_	0.058*** *p*=0.0000	−0.105*** *p*=0.0000	0.148*** *p*=0.0000	0.354*** *p*=0.0000	1	−0.162*** *p*=0.0000	−0.148*** *p*=0.0000	0.121*** *p*=0.0000
*Cash*_*i*,*t*_	−0.033*** *p*=0.0001	0.031*** *p*=0.0002	-0.002 *p*=0.8404	0.087*** *p*=0.0000	−0.073*** *p*=0.0000	1	0.073*** *p*=0.0000	0.045*** *p*=0.0000
*Hold*_*i*,*t*_	0.090*** *p*=0.0000	0.210*** *p*=0.0000	−0.287*** *p*=0.0000	−0.156*** *p*=0.0000	−0.201*** *p*=0.0000	0.042*** *p*=0.0000	1	−0.071*** *p*=0.0000
*Board*_*i*,*t*_	−0.060*** *p*=0.0000	−0.098*** *p*=0.0000	0.195*** *p*=0.0000	0.340*** *p*=0.0000	0.135*** *p*=0.0000	0.037*** *p*=0.0000	−0.129*** *p*=0.0000	1

### The test of geographical peer effect in serial M&A

According to H1, Table 4 is the test result of whether there is a geographical peer effect in the corporate serial M&A. Among them, column (1) tests the influence of control variables on the serial M&A of the focal firm, and the regression coefficient shows that the asset size, leverage level and executive shareholding have a significant and positive impact on corporate serial M&A, while cash flow and board size have a significant negative impact on corporate serial M&A. Column (2) only tests the impact of the main explanatory variable, the average value of serial M&A of geographical peers, on serial M&A of the focal firm. The results show that the estimated coefficient is 0.358 (*t* = 7.25), which is significant at the 1% level, supporting H1. According to model (1), the estimated coefficient shown by the test results in column (3) is 0.357 (*t* = 7.19), which is significant at the 1% level, indicating that the average value of serial M&A of peer firms increased by 10 percentage points, and the number of serial M&A of focal firm will increase by 3.57%, indicating that the average value of serial M&A of geographical peer firm has a significant positive impact on the serial M&A of the focal firm, that is, there has geographical peer effect in the serial M&A of a firm, which supports H1.

**Table 4:** The test of geographical peer effect in serial M&A.

*Variable*	(1)*SMA*_*i*,*t*_		(2)*SMA*_*i*,*t*_		(3)*SMA*_*i*,*t*_	
	coef.	t-value	coef.	t-value	coef.	t-value
$\bar{{SMA}_{-i,t}^{\alpha }}$			0.358***	7.25	0.357***	7.19
			*p*=0.000		*p*=0.000	
*Size*_*i*,*t*_	0.089***	6.39			0.093***	6.65
	*p*=0.000				*p*=0.000	
*Lev*_*i*,*t*_	0.537***	7.91			0.540***	7.96
	*p*=0.000				*p*=0.000	
*Cash*_*i*,*t*_	−0.405***	-2.85			−0.403***	-2.85
	*p*=0.004				*p*=0.004	
*Hold*_*i*,*t*_	1.074***	8.06			1.001***	7.53
	*p*=0.000				*p*=0.000	
*Board*_*i*,*t*_	−0.035***	-4.47			−0.034***	-4.42
	*p*=0.000				*p*=0.000	
*Ind*	*Control*		*Control*		*Control*	
*Year*	*Control*		*Control*		*Control*	
*Cons*	−1.468***	-5.01	0.144	1.52	−1.749***	-5.83
	*p*=0.000		*p*=0.128		*p*=0.000	
*Obs*	14 519		14 519		14 519	
*R* ^2^	0.102 9		0.093 4		0.106 4	

### The test of the impact of social learning

According to H2, Table 5 tests the impact of social learning on the geographical peer effect of serial M&A. According to model 2, columns (1)-(3) test the impact of the leading peers’ average serial M&A on backward focal firm’s serial M&A. Column (1) examines the effect of the control variables on the decision-making of backward focal firm’s serial M&A. Column (2) tests the impact of the leading peers’ average serial M&A on the decision-making of backward focal firm’s serial M&A. The results show that the estimated coefficient of $\bar{SMAtop_{-i,t}^{\alpha }}$ is 0.038 (*t*=1.91), which is significant at the 10% level, supporting H2a. According to model 2, the test results in column (3) show that the estimated coefficient of $\bar{SMAtop_{-i,t}^{\alpha }}$ is 0.045 (*t*=2.20), which is significant at the 10% level, indicating that when the leading peers’ average serial M&A increased by 10 per cent points, the decision-making of backward focal firm’s serial M&A will increase by 0.45%. It can be seen that the formation of the geographical peer effect of corporate serial M&A is at least partly due to the rational social learning of “leading” peer firms by “backward” focal firms, which supports H2a. Similarly, column(4)-(6) tests the impact of the backward peers’ average serial M&A on leading focal firm’s serial M&A. Column (4) examines the impact of the main control variables on the decision-making of leading focal firm’s serial M&A. Column (5) examines the impact of the backward peers’average serial M&A on the decision-making of the leading focal firm’s serial M&A, and the results show that the estimated coefficient of $\bar{SMAbot_{-i,t}^{\alpha }}$ is 0.097(*t*=1.55, *p*=0.122), which does not pass the significant test. According to model 3, the estimated coefficient of $\bar{SMAbot_{-i,t}^{\alpha }}$ in column (6) is 0.094(*t*=1.51, *p*=0.132), which also does not pass the significant test. It shows that the decision-making of leading focal firm’s serial M&A is not significantly affected by the backward peers’ average serial M&A, and does not support H2b. It can be seen that the imitation of the serial M&A decision-making of geographical peer firms by focal firms is not a blind conformity behavior, but a learning behavior based on the acquisition and analysis of geographical peer information. Because the maturity, influence and status advantage of the leading firms are far greater than those of the backward firms, the information value of the serial M&A decision-making of the leading firms is higher, and the autonomy of the serial M&A decision-making of the leading firms is stronger. Therefore, the leading firms are less dependent on the information of other peer firms when making serial M&A decisions.

**Table 5:** The test of the impact of social learning.

*Variable*	$\left(1\right)SMAbot_{i,t}^{\alpha }$		$\left(2\right)SMAbot_{i,t}^{\alpha }$		$\left(3\right)SMAbot_{i,t}^{\alpha }$		$\left(4\right)SMAtop_{i,t}^{\alpha }$		$\left(5\right)SMAtop_{i,t}^{\alpha }$		$\left(6\right)SMAtop_{i,t}^{\alpha }$	
	coef.	t-value	coef.	t-value	coef.	t-value	coef.	t-value	coef.	t-value	coef.	t-value
$\bar{SMAtop_{-i,t}^{\alpha }}$			0.038*	1.91	0.045**	2.20						
			*p*=0.057		*p*=0.028							
$\bar{SMAbot_{-i,t}^{\alpha }}$									0.097	1.55	0.094	1.51
									*p*=0.122		*p*=0.132	
*Size*_*i*,*t*_	0.061***	5.23			0.064***	5.41	0.091***	5.04			0.091***	5.05
	*p*=0.000				*p*=0.000		*p*=0.000				*p*=0.000	
*Lev*_*i*,*t*_	0.436***	6.63			0.440***	6.68	0.475***	6.03			0.475***	6.02
	*p*=0.000				*p*=0.000		*p*=0.000				*p*=0.000	
*Cash*_*i*,*t*_	−0.319***	-2.77			−0.323***	-2.79	−1.240***	-5.66			−1.290***	-5.66
	*p*=0.006				*p*=0.005		*p*=0.000				*p*=0.000	
*Hold*_*i*,*t*_	1.040***	7.91			1.027***	7.82	1.256***	8.24			1.253***	8.23
	*p*=0.000				*p*=0.000		*p*=0.000				*p*=0.000	
*Board*_*i*,*t*_	−0.0160**	-2.06			−0.015*	-1.93	−0.042***	-5.13			−0.042***	-5.16
	*p*=0.040				*p*=0.054		*p*=0.000				*p*=0.000	
*Ind*	*Control*		*Control*		*Control*		*Control*		*Control*		*Control*	
*Year*	*Control*		*Control*		*Control*		*Control*		*Control*		*Control*	
*Cons*	−0.955***	-3.87	0.359***	3.85	−1.036***	-4.12	−1.446***	-3.84	0.254**	2.42	−1.495***	-4.02
	*p*=0.000		*p*=0.000		*p*=0.000		*p*=0.000		*p*=0.016		*p*=0.000	
*Obs*	13 118		13 118		13 118		13 099		13 099		13 099	
*R* ^2^	0.089 9		0.078 9		0.090 3		0.113 4		0.099 8		0.113 6	

### The test of the impact of director network

In response to hypothesis 3, Table 6 tests the moderating effect of director network relationships on the geographical peer effect of serial M&A of firms according to model (4). Column (1) tests the effect of the moderating variable director network relationship *Network*_*i*,*t*_ and the main control variable on the number of serial M&As *SMA*_*i*,*t*_ of the focal firm, and the results indicate that the director network relationship is also an explanatory variable for serial M&A of the focal firm. Column (2) examines only the effect of geographical peer effect $\bar{{SMA}_{-i,t}^{\alpha }}$, director network relationship *Network*_*i*,*t*_ and the cross-product term of the two $\bar{{SMA}_{-i,t}^{\alpha }}\times Network_{i,t}$ on serial M&A of focal firms and the results show an estimated coefficient of $\bar{{SMA}_{-i,t}^{\alpha }}\times Network_{i,t}$ is 0.214 (*t* = 1.64), which is significant at the 10% level which supports hypothesis 3. According to the test results in column (3) of the model (4), the estimated coefficientis $\bar{{SMA}_{-i,t}^{\alpha }}\times Network_{i,t}$ is 0.223 (t = 1.72), which is significant at the 10% level, indicating that the director network relationship *Network*_*i*,*t*_ enhances the positive effect of geographical peer effect on serial M&A of focal firms. In other words, the director network relationship *Network*_*i*,*t*_ plays a significant positive moderating role in the relationship between the mean value of serial M&A of geographic peer firms and serial M&A of focal firms. That is, the richer the structural hole of the director network, the greater the number of serial M&A of focal firms is influenced by the geographic peer effect, thus giving support to hypothesis 3.

**Table 6:** The test of the impact of director network.

*Variable*	(1)*SMA*_*i*,*t*_		(2)*SMA*_*i*,*t*_		(3)*SMA*_*i*,*t*_	
	coef.	t-value	coef.	t-value	coef.	t-value
$\bar{{SMA}_{-i,t}^{\alpha }}\times Network_{i,t}$			0.214*	1.69	0.223*	1.72
			*p*=0.100		*p*=0.086	
$\bar{{SMA}_{-i,t}^{\alpha }}$			0.210**	2.01	0.189*	1.81
			*p*=0.100		*p*=0.070	
*Network*_*i*,*t*_	0.611***	8.88	0.242*	1.71	0.332**	2.33
	*p*=0.000		*p*=0.087		*p*=0.020	
*Size*_*i*,*t*_	0.082***	5.87			0.085***	6.10
	*p*=0.000				*p*=0.000	
*Lev*_*i*,*t*_	0.505***	7.43			0.509***	7.49
	*p*=0.000				*p*=0.000	
*Cash*_*i*,*t*_	−0.405***	-2.94			−0.400***	-2.92
	*p*=0.003				*p*=0.004	
*Hold*_*i*,*t*_	1.324***	9.59			1.261***	9.12
	*p*=0.000				*p*=0.000	
*Board*_*i*,*t*_	−0.044***	-5.63			−0.043***	-5.49
	*p*=0.000				*p*=0.000	
*Ind*	*Control*		*Control*		*Control*	
*Year*	*Control*		*Control*		*Control*	
*Cons*	−1.647***	-5.61	-0.031	-0.23	−1.719***	-5.60
	*p*=0.000		*p*=0.819		*p*=0.000	
*Obs*	14 519		14 519		14 519	
*R* ^2^	0.107 1		0.096 8		0.110 8	

## Robustness test

### Change model

At first, to test whether there is a nonlinear relationship between the focal firm and the geographical peer effect, the squared term of the geographical peer effect is added to the baseline model (1), as in column (1) of Table 7. The estimated coefficients ${\bar{{SMA}_{-i,t}^{\alpha }}}^{2}$ shown in the results of column (1) are not significant. In contrast the primary term coefficients $\bar{{SMA}_{-i,t}^{\alpha }}$ are significant at the 1% level, revealing that the model of the impact of geographical peer effects on serial M&A of firms is basically acceptable for the setting of model (1). Next, to avoid endogeneity problems due to omitted variables, the control variables of peer firms are included in the previous model relative to the control variables of the focal firm, i.e., the average firm size of geographic peer firms, the average leverage level of geographic peer firms, the average cash flow of geographic peer firms, the average executive ownership level of geographic peer firms, and the average board size of geographic peer firms, as shown in columns (2) and (3) of Table 4. The results indicate that including the peer firm control variables still supports hypotheses 1 and 3. Because the focal firm’s serial M&A belong to the censored data with the left side limited point of 0, we use the Tobit model to re-test the baseline model in the previous paper, and the results are shown in column (4) of the table. The estimated coefficient $\bar{{SMA}_{-i,t}^{\alpha }}$ of is 1.393 (*t*=24.36), which is significantly positive at the 1% level, and in line with the previous paper.

**Table 7:** The robustness test of change model and variables.

*Variable*	Nonlinear test						Tobit test		Change variables					
	(1)*SMA*_*i*,*t*_		(2)*SMA*_*i*,*t*_		(3)*SMA*_*i*,*t*_		(4)*SMA*_*i*,*t*_		(5)*SMA*_*i*,*t*_		(6)*SMA*_*i*,*t*_		(7)*SMA*_*i*,*t*_	
	coef.	t-value	coef.	t-value	coef.	t-value	coef.	t-value	coef.	t-value	coef.	t-value	coef.	t-value
${\bar{{SMA}_{-i,t}^{\alpha }}}^{2}$	-0.033	-0.59												
$\bar{{SMA}_{-i,t}^{\alpha }}$	0.447***	2.93	0.242***	4.54	0.210*	1.70	1.393***	24.36	0.932***	5.50	0.856***	11.87	0.241***	3.77
*Netowrk*_*i*,*t*_					0.349**	2.46								
$\bar{{SMA}_{-i,t}^{\alpha }}\times Netowrk_{i,t}$					0.210*	1.70								
*Controls*	*Control*		*Control*		*Control*		*Control*		*Control*		*Control*		*Control*	
*Ind*	*Control*		*Control*		*Control*		*Control*		*Control*		*Control*		*Control*	
*Year*	*Control*		*Control*		*Control*		*Control*		*Control*		*Control*		*Control*	
*Cons*	−1.785***	-5.77	2.350***	3.22	2.313***	3.17	-4.024	-9.61	−8.158***	-5.37	−5.915***	-4.38	−1.591***	-5.34
*Obs*	14 519		14 519		14 519		14 519		1 763		1 763		14 519	
*R* ^2^	0.106 4		0.109 0		0.113 4		0.020 4		0.240 6		0.194 7		0.107 4	

### Change variables

Change the measurement of the geographic peer effect of the explanatory variable, as shown in columns (5) and (6) of Table 7. Select the focal firm whose registered address and office address are inconsistent as the sample; column (5) uses the registered place as the peer division standard, and column (6) uses the office address as the peer division standard. The results support H1. Change the measure of the moderator variable, as shown in column (7) of Table 7. Referring to Larcker, So and Wang (2013) [102], director network centrality is used to measure the director network, and the results support H3.

### Lag test

Columns (1)-(3) of Table 8, on the basis of the above, conduct a robustness test on the one-period lag of explanatory variables, moderator variables and control variables. The results support H1, H2a and H3.

**Table 8:** The robustness test of lag one-period and IV.

*Variable*	(1)*SMA*_*i*,*t*_		(2)*SMA*_*i*,*t*_		(3)*SMA*_*i*,*t*_		(4)*SMA*_*i*,*t*_		(5)*SMA*_*i*,*t*_		(6)*SMA*_*i*,*t*_		(7)*SMA*_*i*,*t*_	
	coef.	t-value	coef.	t-value	coef.	t-value	coef.	t-value	coef.	t-value	coef.	t-value	coef.	t-value
$\bar{{SMA}_{-i,t-1}^{\alpha }}$	0.297***	4.98			0.153	1.17								
$\bar{SMAtop_{-i,t-1}^{\alpha }}$			0.036**	2.05										
*Netowrk*_*i*,*t*−1_					-0.079	-0.45								
$\bar{{SMA}_{-i,t-1}^{\alpha }}\times Netowrk_{i,t-1}$					0.225*	1.38								
$\bar{{SMA}_{-i,t}^{\alpha }}$							1.138***	17.46			4.335***	19.10	0.043*	1.78
$\bar{SMAtop_{-i,t}^{\alpha }}$									0.956***	29.52				
*Netowrk*_*i*,*t*_											6.116***	18.06		
$\bar{{SMA}_{-i,t}^{\alpha }}\times Netowrk_{i,t}$											4.482***	16.01		
*Controls*	*Control*		*Control*		*Control*		*Control*		*Control*		*Control*		*Control*	
*Ind*	*Control*		*Control*		*Control*		*Control*		*Control*		*Control*		*Control*	
*Year*	*Control*		*Control*		*Control*		*Control*		*Control*		*Control*		*Control*	
*Cons*	−1.417***	-4.41	−1.217***	-4.67	-1.812	-5.50	0.112	1.55	−1.463***	-6.28	−5.798***	-13.93	2.425***	49.47
*Obs*	12 819		12 819		12 819		14 519		13 118		14 519		7 154	
*R* ^2^	0.103 7		0.089 4		0.109 9								0.0061	
First-stage IV							0.874***		0.989***		0.661***			
Wald Chi2							1448.74***		1084.03***		1650.16***			

### IV test

Refer to Zhang, Yao and Du (2021) [32] with “peers of peers” as an instrumental variable. Specifically, if two sets of peer firm groups that are not completely coincident are intersected, the non-intersecting parts of the two sets can be valid tool variables for each other. The mathematical set can be used to express the peer firm relationship: let C=*A* ⋒ *B*(*C* ≠⌀ and *C* ≠A, *C* ≠B), for the focal firm *i*, the peer firm set is E={*x* ∣ *x* ∈ *B* *and* *x* ∉ *C*} and the total peers of peers of the focal firm is F={*y* ∣ *y* ∈ *A and y* ∉ *C*}. The measurement method of the instrumental variable is specifically to randomly select any firm k from other peers in the same region as the focal firm, and then randomly select a firm m from the group of peer firms in the same region as the firm k, then the average number of serial M&A of the peer firm m can be used as an instrumental variable for the geographical peer effect of the focal firm, and the 2SLS regression results are shown in columns (5)-(6) of Table 8. The results show that instrumental variables are effective and support H1, H2a and H3.

### COVID -19 pandemic samples test

Considering that the relationship between geographical peer effect and serial M&A decision-making of focal firm may change during or after the COVID -19 pandemic, this study intends to use sample data from the COVID—19 period from 2020 to 2022 to test the robustness of the geographic peer effect in serial M&A, as shown in column (7) of Table 8. The results support H1.

## Discussion

### Heterogeneity test of focal firm’s serial M&A

Manski(2000) believed that an organization or individual making consistent behavioral decisions concerning other group members is a passive and mechanical response to the external environment [87]. In other words, the focal firm’s serial M&A and the peer firms’ average serial M&A are symmetrical in scope and direction. According to the previous analysis of information-based theory, rivalry-based theory, culture and language, the geographical peer effect of serial M&A is a complex response mechanism driven by rationality. Shroff, Verdi and Yost (2017) have pointed out that when focal firm information is scarce, peer information plays an important role in reducing information asymmetry. However, with the increase of the amount of focal firm information, this effect weakens [103]. Therefore, the focal firm’s serial M&A are divided into four sub-samples to observe the asymmetry of the influence of the peers’average serial M&A on the direction and scope of focal firm’s serial M&A. Column(1) to (4) of Table 9 are the heterogeneity test of focal firm’s serial M&A. It can be seen from column (1) that when *SMA*_*i*,*t*_ > = 1, the estimated coefficient of $\bar{{SMA}_{-i,t}^{\alpha }}$ is 0.319, which is significant at 1% level. In column (2), when *SMA*_*i*,*t*_ > = 2, the estimated coefficient of $\bar{{SMA}_{-i,t}^{\alpha }}$ is 0.211, which is significant at 10% level. In column (3) and (4), when *SMA*_*i*,*t*_ > = 3 and *SMA*_*i*,*t*_ > = 4, the estimated coefficient of $\bar{{SMA}_{-i,t}^{\alpha }}$ is 0.254 and 0.099, not significant. Compared with column (3), the estimated coefficient value of column (4) is less. The results of columns (1)-(4) show that with the increase of the number of focal firm’s serial M&A, the value and significance of the estimated coefficient of peers’average serial M&A $\bar{{SMA}_{-i,t}^{\alpha }}$ are gradually decreasing. It indicates that the less of focal firm’s serial M&A, the more susceptible to the influence of geographical peer effect. It can be seen that the geographical peer effect of serial M&A decision-making is not the mechanical passive response of focal firms to other peers, but a complex reaction mechanism driven by reason. When the focal firm has less decision-making information about serial M&A, the geographical peer information can reduce the information asymmetry, and the geographical peer effect has a significant impact on the serial M&A decision-making. With the increase of serial M&A decision-making information of focal firms, the influence of geographical peer effect is weakened.

**Table 9:** The heterogeneity test of serial M&A frequency of focal firms and peer firms.

*Variable*	(1)*SMA*_*i*,*t*_ ≥ 1		(2)*SMA*_*i*,*t*_ ≥ 2		(3)*SMA*_*i*,*t*_ ≥ 3		(4)*SMA*_*i*,*t*_ ≥ 4		(5)*SMA*_*i*,*t*_		(6)*SMA*_*i*,*t*_		(7)*SMA*_*i*,*t*_		(8)*SMA*_*i*,*t*_	
	coef.	t-value	coef.	t-value	coef.	t-value	coef.	t-value	coef.	t-value	coef.	t-value	coef.	t-value	coef.	t-value
$\bar{{SMA}_{-i,t-1}^{\alpha }}$	0.319***	4.37	0.211*	1.82	0.254	1.45	0.099	0.38								
$\bar{{SMA}_{-i,t}^{\alpha }\leq 0.5}$									-0.606	-1.28						
$0.5<\bar{{SMA}_{-i,t}^{\alpha }<1}$											0.424***	2.66				
$1\leq \bar{{SMA}_{-i,t}^{\alpha }<2}$													0.791***	6.88		
$\bar{{SMA}_{-i,t}^{\alpha }\geq 2}$															-0.324	-0.66
*Controls*	*Control*		*Control*		*Control*		*Control*		*Control*		*Control*		*Control*		*Control*	
*Ind*	*Control*		*Control*		*Control*		*Control*		*Control*		*Control*		*Control*		*Control*	
*Year*	*Control*		*Control*		*Control*		*Control*		*Control*		*Control*		*Control*		*Control*	
*Cons*	−1.894***	-4.18	−2.109***	-2.74	−3.222***	-2.52	−4.787***	-2.24	0.455	0.95	-0.446	-1.25	−3.748***	-6.13	−2.283*	-1.66
*Obs*	7250		3699		2037		1187		1 555		5116		6921		927	
*R* ^2^	0.1001		0.0991		0.1031		0.1132		0.043 9		0.032 7		0.099 3		0.136 9	

### Heterogeneity test of peers’average serial M&A

Furthermore, the peers’average serial M&A are divided into four sub-samples in columns(5) to (8) of Table 9 to observe the asymmetry of the influence of the peers’ average serial M&A on the direction and scope of the focal firm’s serial M&A. It can be seen from column (5) that when $\bar{{SMA}_{-i,t}^{\alpha }}\leq 0.5$, the estimated coefficient of peers’average serial M&A $\bar{{SMA}_{-i,t}^{\alpha }}$ is -0.606, which is not significant. It shows that when the average serial M&A of peer firms do not exceed once every two years, the geographical peer effect has no significant impact on the serial M&A decision-making of the focal firm. In columns (6) and (7), when $0.5<\bar{{SMA}_{-i,t}^{\alpha }}<1$ and $1\leq \bar{{SMA}_{-i,t}^{\alpha }}<2$, the estimated coefficient of $\bar{{SMA}_{-i,t}^{\alpha }}$ is 0.424 and 0.791, which are both significant at the 1% level. It shows that when the peer firms have 0.5 to 2 serial M&A per year on average, the geographical peer effect has a significant positive impact on the decision-making of serial M&A of the focal firm. Compared with column (6), the estimated coefficient value of column (7) is larger. It shows that within a specific range, the larger the peers’average serial M&A, the greater the effect of the geographical peer effect in the decision-making of the focal firm’s serial M&A. In column (8), When $\bar{{SMA}_{-i,t}^{\alpha }}\geq 2$, the estimated coefficient of $\bar{{SMA}_{-i,t}^{\alpha }}$ is -0.324, which is not significant. The above results show that the focal firm’s serial M&A decision-making is a complex response to the average serial M&A of peer firms after considering their situation. The influence of geographical peer effect in serial M&A decision-making is the reflection of the focal firms to deal with information asymmetry in the case of uncertain external environment. When the average number of peer firms’ serial M&A is moderate, the focal firms will choose to follow or imitate out of rational considerations, while when the average number of peer firms’ serial M&A is too high or too low, the focal firms will not choose to follow or imitate. The geographical peer effect in serial M&A is a response under rational motivation rather than a simple symmetrical relationship.

### Heterogeneity test of focal firm’s internal learning

The organizational learning mechanism is divided into external and internal learning, and the two have opposite tension [104]. It can be seen from the previous analysis that the peer effect of serial M&A decision-making is a way of external learning. Therefore, it is necessary to investigate the role of internal learning in the influence of geographical peer effect in serial M&A decision-making. Hayward (2002) [20] has believed that the serial M&A interval of 6–12 months is conducive to generating learning behavior. The M&A interval is the time between the completion of the M&A transaction and the announcement date of the next M&A transaction. Because of this, the serial M&A interval is used to measure internal learning (*EXP*_*i*,*t*_). When the M&A interval of serial M&A is more than half a year (180 days), the value of *EXP*_*i*,*t*_ is 1, indicating that the focal firm has learned the past serial M&A experience adequately. Otherwise it takes the value of 0. Table 10 shows the impact of internal learning on the geographical peer effect in serial M&A. Where column (1) is a full-sample test of the moderating effect of internal learning. Columns (2) and (3) are the group test that the focal firms have or not have adequately learned the serial M&A experience respectively. The results of column (1) show that the estimated coefficient of $\bar{{SMA}_{-i,t}^{\alpha }}\times {EXP}_{i,t}$ is -0.245 (*t*=-3.93), which is significant at the 1% level, and internal learning weakens the positive impact of geographical peer effects on the focal firm’s serial M&A decision-making. Columns (2) and (3) show a relative synergy between internal learning and peer effect. First of all, the focal firm takes into account both internal learning and peer learning [105]. Whether internal learning is high or low, it is significantly positively affected by the peer effect. Secondly, internal learning and learning from peers are in a state of imbalance. When internal learning is higher, the peer effect has less influence on the decision-making of serial M&A (0.202). When the internal learning is low, the peer effect has a more significant influence on the decision-making of serial M&A (0.375). The negative moderating effect of internal learning on geographical peer effect also shows that the influence of geographical peer effect on serial M&A decision-making is the rational response of focal firms to peer firm decision-making information acquisition and analysis. When the internal learning ability of the firm is strong, the impact of serial M&A decisions of geographical peer firms on the decision-making of focal firms is weakened.

**Table 10:** The test of the adequate internal learning.

*Variable*	(1)*SMA*_*i*,*t*_		(2)*SMA*_*i*,*t*_		(3)*SMA*_*i*,*t*_	
	coef.	t-value	coef.	t-value	coef.	t-value
$\bar{{SMA}_{-i,t}^{\alpha }}\times {EXP}_{i,t}$	−0.245***	-3.93				
$\bar{{SMA}_{-i,t}^{\alpha }}$	0.396***	6.85	0.202***	3.12	0.375***	5.72
*EXP*_*i*,*t*_	1.302***	17.40				
*Controls*	*Control*		*Control*		*Control*	
*Ind*	*Control*		*Control*		*Control*	
*Year*	*Control*		*Control*		*Control*	
*Cons*	−1.817***	-6.11	0.023***	0.06	−2.114***	-5.53
*Obs*	14 519		4 137		10 382	
*R* ^2^	0.156 9		0.049 1		0.110 7	

## Conclusions

Based on the serial M&A data of Chinese listed companies from 2010 to 2019, this paper studies whether there have geographical peer effects in corporate serial M&A and the impact of social learning and director networks on this effect. The research finds that there are geographical peer effects in the corporate serial M&A, and the geographical peer firms’ average serial M&A decision-making has a significant positive impact on the serial M&A decision-making of the focal firm. The social learning of “backward” focal firms to the serial M&A decision-making of “leading” peer firms is an important factor for the geographical peer effect, not vice versa. It can be seen that the social learning of the geographical peer effect of serial M&A is directional, and one of the mechanisms of the regional peer effect is derived from the rational learning of the serial M&A decision-making of “leading” peer firms. The director network mechanism shows that the richer the structural holes of the director network relationship, the stronger the positive influence of the geographical peer effect in serial M&A. The structural hole of the director network relationship positively moderates the relationship between geographical peer effect and corporate serial M&A decision-making; the richer the director network relationship, the stronger the geographical peer effect of serial M&A decision-making.

Further analysis finds that in the case where the number of serial M&A of the focal firm and the average number of serial M&A of peer firms are different, there are differences in the geographical peer effect in serial M&A. Firstly, the influence of the geographical peer effect decreases with the increase of the number of focal firm’s serial M&A. When the focal firm’s serial M&A is not less than 3, the geographical peer effect of serial M&A is not significant. Secondly, only when the average number of geographical peer firms’ serial M&A is within a reasonable range will the focal firm imitate the decision-making of peer firms, thereby producing the geographical peer effect in serial M&A. When the peer firms’ average serial M&A is higher than 2 or lower than 0.5, the uncertainty caused by following the behavior of the peer firms will increase, and imitating the decision-making of peer firms’ serial M&A may not be conducive to reducing risks or obtaining legitimacy, resulting in the insignificant geographical peer effect in serial M&A. In addition, adequate internal learning of the focal firm’s serial M&A decision-making will reduce its imitation of geographical peer firms’ decision-making. However, in order to reduce the cost and risk of decision-making, the focal firms without adequate internal learning are more inclined to imitate the decision-making of peer firms to produce a geographical peer effect.

This study provides the following enlightenment for the formulation and optimization of the corporate serial M&A decision-making: Firstly, this paper studies the corporate serial M&A decision-making from the perspective of geographical peer effect, breaking through the bottleneck of most previous literature that only analyzes the serial M&A decision-making from the corporate itself. The research results show that firms should pay attention to the geographical peer effect, and employ it to make reasonable serial M&A decision-making. In the process of M&A transactions, firms are faced with problems such as information asymmetry and risk uncertainty. Imitating the serial M&A decision-making of geographical peer firms may help reduce the problems such as information asymmetry and risk uncertainty. Secondly, it finds that social learning and director network play an important role in the relationship between corporate serial M&A and geographical peer effect, both promoting the positive impact of the geographical peer effect in serial M&A decision-making. Among them, the social learning behavior mainly comes from learning the geographical “leading” peers rather than the “backward” peers. It can be seen that the peer effect is different from the “herd effect” and has an upward direction and scientific rationality. Therefore, firms must conduct a comprehensive understanding and analysis of the peer effect in serial M&A. Thirdly, in order to improve the efficiency of serial M&A decision-making, regulatory authorities should reasonably guide the impact of geographical peer effects in serial M&A, and give full play to the geographical peer effect in serial M&A to improve the efficiency of corporate M&A decision-making. They should pay attention to the implementation of serial M&A strategies of “leading” firms and firms with rich director network relationships, and give full play to the positive impact of peer effects in serial M&A through typical demonstrations and policy guidance. For M&A projects that promote technological innovation, green development, and social responsibility, regulators can use the conduction and diffusion of peer effects to promote serial M&A decision-making when formulating M&A policies. For restrictive M&A projects, the regulatory authorities should strengthen their review efforts to prevent systemic risks caused by the spread of serial M&A peer effects.

Although this paper has a certain contribution to the influence of geographical peer effect in the formation of serial M&A decision-making, there are still the following shortcomings, which need to be further discussed in future research. First of all, this paper takes the geographical peer enterprises as the reference group, takes the regional peer firms’ serial M&A average decision-making as the agent variable, and the peer effect model uses the mean linear model in the reference group as the identification condition, but the spatial model and discrete selection model can also be used as the identification conditions of peer effect. In the future, we can continue to use the spatial model and discrete selection model to identify the impact of geographical peer effect in serial M&A decision-making. Secondly, this paper only discusses the influence of learning behavior and director network on geographical peer effect and serial M&A decision-making relationship. however, external environments such as product market competition and economic policy uncertainty will also cause enterprises to choose to imitate or follow peer firms’ decision-making. In the future, we can continue to test the influence mechanism of the external environment on serial M&A decision-making.

## Supporting information

S1 Data Geographical peer effect in serial M&A and director network. (XLSX)

S2 Data Geographical peer effect social learning-top10 to other’s affect. (XLSX)

S3 Data Geographical peer effect social learning-bot10 to top90. (XLSX)
